# Adsorption and Thermal Decomposition of Triphenyl Bismuth on Silicon (001)

**DOI:** 10.1021/acs.jpcc.3c03916

**Published:** 2023-08-14

**Authors:** Eric A. S. Lundgren, Carly Byron, Procopios Constantinou, Taylor J. Z. Stock, Neil J. Curson, Lars Thomsen, Oliver Warschkow, Andrew V. Teplyakov, Steven R. Schofield

**Affiliations:** † London Centre for Nanotechnology, University College London, WC1H 0AH London, U.K.; ‡ Department of Physics and Astronomy, University College London, WC1E 6BT London, U.K.; § Department of Chemistry and Biochemistry, University of Delaware, Newark, Delaware 19716, United States; ∥ Paul Scherrer Institute, 5232 Villigen, Switzerland; ⊥ Department of Electronic and Electrical Engineering, University College London, WC1E 7JE London, U.K.; # Australian Synchrotron, ANSTO, Clayton, Victoria 3168, Australia

## Abstract

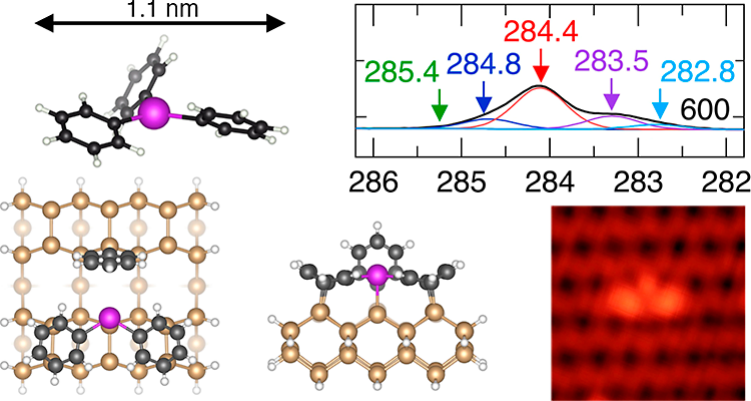

We investigate the adsorption and thermal decomposition of triphenyl bismuth (TPB) on the silicon (001) surface using atomic-resolution scanning tunneling microscopy, synchrotron-based X-ray photoelectron spectroscopy, and density functional theory calculations. Our results show that the adsorption of TPB at room temperature creates both bismuth–silicon and phenyl–silicon bonds. Annealing above room temperature leads to increased chemical interactions between the phenyl groups and the silicon surface, followed by phenyl detachment and bismuth subsurface migration. The thermal decomposition of the carbon fragments leads to the formation of silicon carbide at the surface. This chemical understanding of the process allows for controlled bismuth introduction into the near surface of silicon and opens pathways for ultra-shallow doping approaches.

## Introduction

A thorough understanding of the chemisorption and dissociation of precursor molecules on semiconductor surfaces is crucial for precisely controlling the selective incorporation of dopant atoms and the fabrication of ultra-shallow doping profiles in semiconductors. Existing methods for introducing large dopants, like bismuth, typically involve the thermal diffusion of the dopants from a layer of solid material deposited on the surface,^1,2^ or the kinetic implantation of dopants into the substrate.^3^ However, new applications in microphotonics^4^ and spintronics^5^ have highlighted the need for more precise control over the concentration and location of bismuth atoms in target substrates, as well as a deeper understanding of the quantum mechanical properties of bismuth-doped semiconductors, particularly silicon.^6−8^ While there has been progress toward the deterministic control of bismuth ion implantation into silicon,^9^ such approaches are unlikely to achieve the sub-nanometer precision that has been demonstrated (both in-plane and out-of-plane) using chemical doping methods for dopants, such as phosphorus, arsenic, and boron.^10−12^

In this article, we explore the potential of using the adsorption and thermal transformation of triphenyl bismuth [Bi(C_6_H_5_)_3_, or TPB] on the silicon (001) surface to control the introduction of bismuth into silicon crystals. TPB is a carbon-based bismuth precursor and as such this work is complementary to our recent investigation of the chlorine-based bismuth precursor, bismuth trichloride (BiCl_3_),^13^ and other recent efforts to evaluate carbon-based precursors for aluminum (trimethyl aluminum and triethyl aluminum).^14^ Using atomic-resolution scanning tunneling microscopy (STM), synchrotron-based X-ray photoelectron spectroscopy (XPS), and density functional theory (DFT) calculations, we show that TPB adsorption at room temperature leads to molecular adsorption via Si–Bi and Si–C bonding, and dissociative adsorption involving the cleaving of a bismuth–phenyl bond. Upon annealing above room temperature, we find complete phenyl detachment at around 140 °C, while bismuth migration into the bulk occurs at higher temperatures of around 360 °C. We observe a 4 × 4 reconstruction, characteristic of silicon-carbide formation,^15^ after annealing to 580 °C. Thus, TPB dosing and surface annealing results in the incorporation and diffusion of bismuth into silicon, presenting a method for the ultra-shallow (nanometer) doping of silicon with bismuth. Despite the fact that this approach introduces carbon impurities onto the surface, it may still prove practical for certain applications because bismuth migrates subsurface while the carbon impurities can be removed from the topmost layer using chemical or physical treatments; e.g., prior work has shown that surface carbon can be removed from Si(001) via oxidation combined with chemical etching^16,17^ or direct physical removal via ion sputtering.^18^ Additionally, it is worth noting that ref (16) provides a detailed exploration of removing carbon above shallow doping layers, complete with electrical transport measurements that affirm the doping layer’s integrity post carbon removal.

## Methods

XPS measurements were performed at the soft X-ray (SXR) beamline of the Australian Synchrotron^19^ in an ultrahigh vacuum (UHV) chamber with a background pressure better than 2 × 10^–10^ mbar. We used a photon energy of 1500 eV, with a pass energy of 10 eV. The positions of the features observed were calibrated against the 84.0 eV peak of Au 4f(7/2). The CasaXPS software^20^ was used to analyze the raw data and the peaks were fitted with a Shirley background and a Gaussian–Lorentzian (70:30) mix.

We obtained atomic resolution STM images at a temperature of 78 kelvin using a commercial Scienta Omicron Low-Temperature STM (LT-STM) in a UHV chamber with background pressure better than 2 × 10^–10^ mbar. For the STM probe, we used chemically etched tungsten tips.

Silicon (001) samples (0.04–0.06 Ω cm, Sb-doped; Virginia Semiconductor Inc.) were degassed at 550 °C overnight, then flash annealed to 1200 °C for 10 s to produce an atomically clean Si(001) surface. Triphenyl bismuth (ChemCruz SC-280160, purity ≥98%) was evaporated onto the clean Si(001) surface using an organic material effusion cell (MBE-komponenten GmbH), with the TPB source heated to 80 °C and the silicon substrate held at room temperature. The chamber background pressure during TPB evaporation was 2 × 10^–9^ mbar.

During sample preparation, the temperature of the samples was monitored using an infrared pyrometer for values above 300 °C. For temperatures ranging between room temperature and 300 °C, we estimated the measurements using a linear extrapolation of the heating power supplied by direct current. This approach allowed us to maintain consistent and accurate temperature control throughout our experiments.

We have performed a DFT survey of possible adsorption structures. Our calculations were performed using the hybrid exact-exchange functional B3LYP,^21,22^ the core-pseudopotential LANL2DZ basis set,^23^ and the Gaussian 16 software.^24^ The LANL2DZ basis set was chosen for its balance of computational efficiency and accuracy, suitable for our aim of exploring a range of possible adsorption structures. Larger basis sets or additional refinements such as zero-point energy corrections (such as those we have employed when studying a smaller bismuth compound on Si(001)^13^) while potentially providing more precise results, would significantly increase the computational cost and time. In the context of this study, where our aim was to explore a broad range of possible adsorption structures rather than definitively assign structures to experimental observations, we deemed the use of the LANL2DZ basis set to be a suitable and pragmatic approach. We note that the LANL2DZ basis set, with its inherent pseudopotential, is suitable for calculations involving bismuth-containing compounds due to its ability to account for relativistic effects. A cluster model of Si_49_H_40_ was employed to depict the Si(001) surface (refer to Supporting Information Figure S2). The chemical termination for all silicon atoms, excluding the surface dimer atoms, consists of 40 hydrogen atoms. To simulate the strain imposed by the neighboring surface and bulk atoms of an extended surface, these hydrogen atoms terminating the cluster were maintained in fixed positions during geometry optimization. Adsorption energies, denoted as *E*_ads_, are calculated in relation to both the clean Si(001) surface and a gaseous TPB molecule
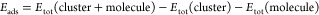
1where *E*_tot_(cluster + molecule) is the total energy of the silicon cluster with an adsorbed molecule, *E*_tot_(cluster) is the total energy of a bare silicon surface cluster, and *E*_tot_(molecule) is the total energy of the gas phase molecule.

## Results and Discussion

### TPB Room-Temperature Adsorption

**Figure 1:**
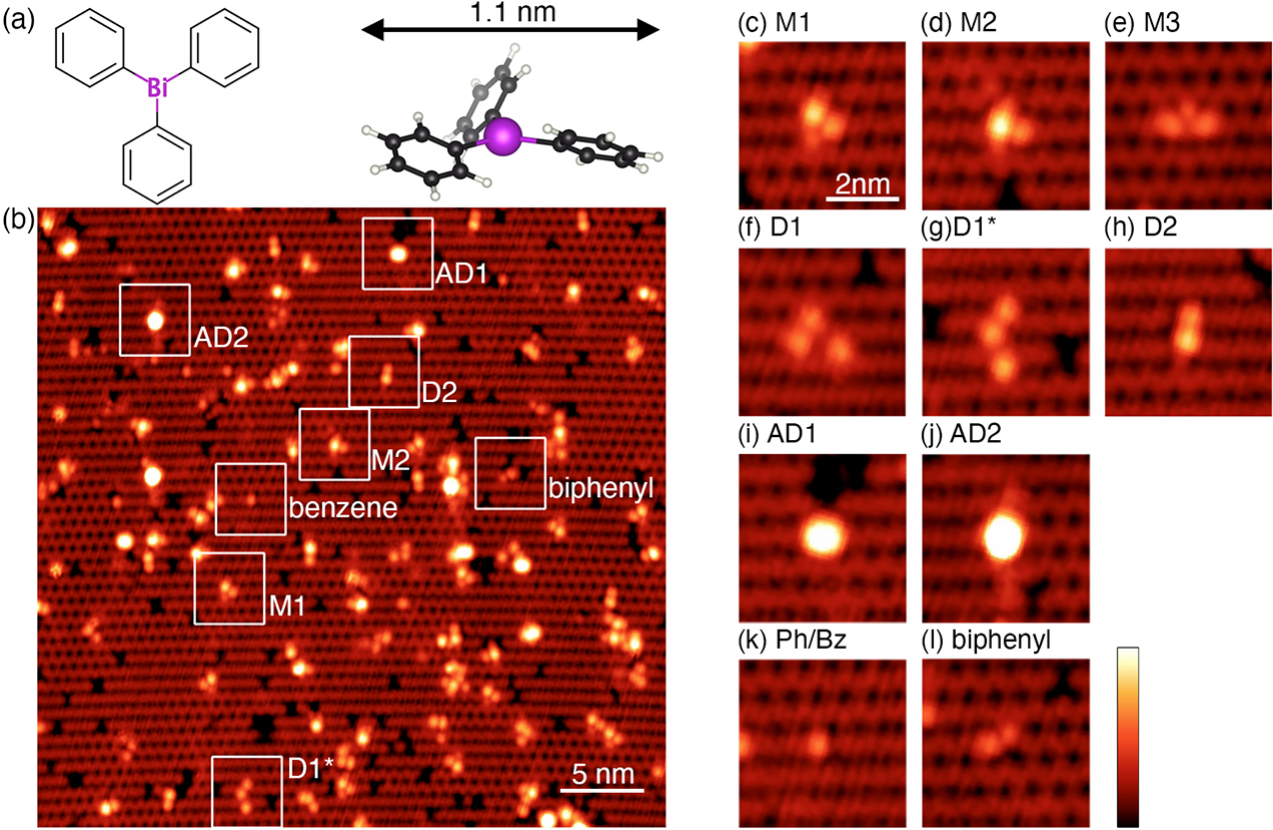
(a) Chemical schematic and ball and stick model of triphenyl bismuth (TPB). (b) STM image of a Si(001) surface exposed to submonolayer coverage of TPB (1 s exposure). (c–h) Individual features attributable to TPB adsorption (see text for description). (i,j) Bi–Bi ad-dimers perpendicular and parallel to the dimer row direction, respectively. (k,l) Features attributable to phenyl/benzene and biphenyl adsorption. Most of the features in panels (c–l) can be found in the overview image in panel (b), and these have been indicated by labeled white squares. See also Supporting Information, Figure S1 that shows images at different TPB coverages. Image parameters: −2 V, 50 pA, and *z*-range 180 pm. (b–l) 4 × 4 nm^2^, 78 K.

Triphenyl bismuth is an organometallic compound consisting of a central bismuth atom bonded to three phenyl rings. A chemical structure model and a ball and stick model of the molecule are shown in Figure 1a. An STM image, acquired at 78 K, of a Si(001) surface after exposure to a sub-monolayer coverage of TPB at room temperature is shown in Figure 1b. The Si(001) surface consists of rows of buckled dimers in a *c*(4 × 2) periodicity; these dimer rows run horizontally in Figure 1b and their periodicity is evident by a zig-zagged appearance.^25^ We see a variety of dark depressions and bright protrusions on the surface. The dark depressions are a combination of vacancy defects and C-defects^26^ that were present on the surface prior to exposure to TPB.

The various bright protrusions seen throughout the image (Figure 1b) were not present before exposure to TPB and can therefore be attributed to the adsorption of TPB. Figure 1c–l provides high-resolution images of the 10 different types of protrusion features that we observe, which represent the most common motifs observed in our study. The first three of these, as shown in Figure 1c–e, and labeled M1, M2, and M3, respectively, each present three bright lobes clustered together within a diameter of ∼1 nm. Comparison to the threefold symmetric structure and dimensions of TPB (Figure 1a) suggests that these features can be attributed to molecularly adsorbed TPB in different bonding configurations on the Si(001) surface. We note that several structures appear to show bonding across the dimer rows. This suggests that the adsorption reaction has occurred not solely within a single dimer row, but that part of the adsorption has occurred across the trough between dimer rows, a phenomenon that, though less common, is known and has been reported in literature.^27,28^

The features shown in Figure 1f,g (labeled D1 and D1*) also present a cluster of three protrusions. However, in these cases, two of the protrusions are close together, while the third protrusion is spatially separated by a distance ≥1 nm. The separation of the third protrusion means that these features cannot be attributed to molecularly adsorbed TPB. Instead, we propose that these features are produced by the dissociative adsorption of TPB, where one of the phenyl rings has dissociated from the main fragment and has become directly attached to a nearby silicon dimer. We classify all such features as D1, and the superscript star in the label of Figure 1g is meant to convey that the location of the third protrusion can vary slightly within this classification.

Another feature is shown in Figure 1h, which we label D2. Unlike the features discussed so far, the D2 feature has only two protrusions. Given the general similarities in its appearance to the D1 features and the above discussion, we suggest that this feature might be assigned to a TPB fragment where one phenyl ring has been dissociated, but where the dissociated phenyl ring is not located close to the adsorbing fragment.

We also find two types of large and bright protrusion features, as shown in Figure 1i,j. These are consistent with surface bismuth ad-dimers.^29,30^ Bismuth ad-dimers on the Si(001) surface have been reported after exposing a clean Si(001) surface to the evaporated elemental bismuth, and also after exposing a clean Si(001) surface to molecular bismuth chloride (BiCl_3_).^13^ In both cases, bismuth ad-atoms form on the surface and rapidly diffuse at room temperature until two bismuth ad-atoms meet and pair up to form a bismuth ad-dimer. The barriers for bismuth diffusion and the pairing of two bismuth ad-atoms to form an ad-dimer have been shown by previous DFT calculations to be less than 0.44 eV, and these processes, therefore, occur rapidly at room temperature.^13,31^ The bismuth ad-dimers are thermally stable at room temperature and do not diffuse on the time scale of a typical STM experiment. The two bismuth ad-dimers shown in Figure 1i,j correspond to configurations where the ad-dimers are on top of the dimer row and aligned parallel (Figure 1i) and perpendicular (Figure 1j) to the dimer rows. The presence of bismuth ad-dimers on the surface suggests that a small fraction of TPB adsorption results in bismuth detachment from the phenyl groups even at room temperature.

Figure 1k,l shows smaller protrusions that by comparison with the literature we attribute to adsorbed phenyl/benzene and bi-phenyl, respectively.^32−34^ The existence of these features on the surface provides an explanation for the missing phenyl ring from structure D2 and the two bismuth ad-dimer features AD1 and AD2.

We have also observed the TPB exposed Si(001) surface as a function of the exposure time. Supporting Information, Figure S1 shows a sequence of STM images highlighting how the TPB adsorbed surface changes as a function of TPB exposure time from 1 to 60 s. We also plot in Supporting Information, Figure S1f, the number of TPB-related features counted within a 2500 nm area of the surface for each exposure and show that it increases linearly for low coverages up to an exposure time of about 20 s. For longer exposure times up to 60 s, the rate at which new features appear becomes slower. No evidence of multilayer formation was observed. Thus, the STM data suggests that TPB molecules initially adsorb easily to the surface but steric hindrance slows the rate of adsorption of additional TPB molecules and the formation of new TPB-related features at higher coverages within the conditions used in this work.

### Density Functional Theory Modeling

**Figure 2:**
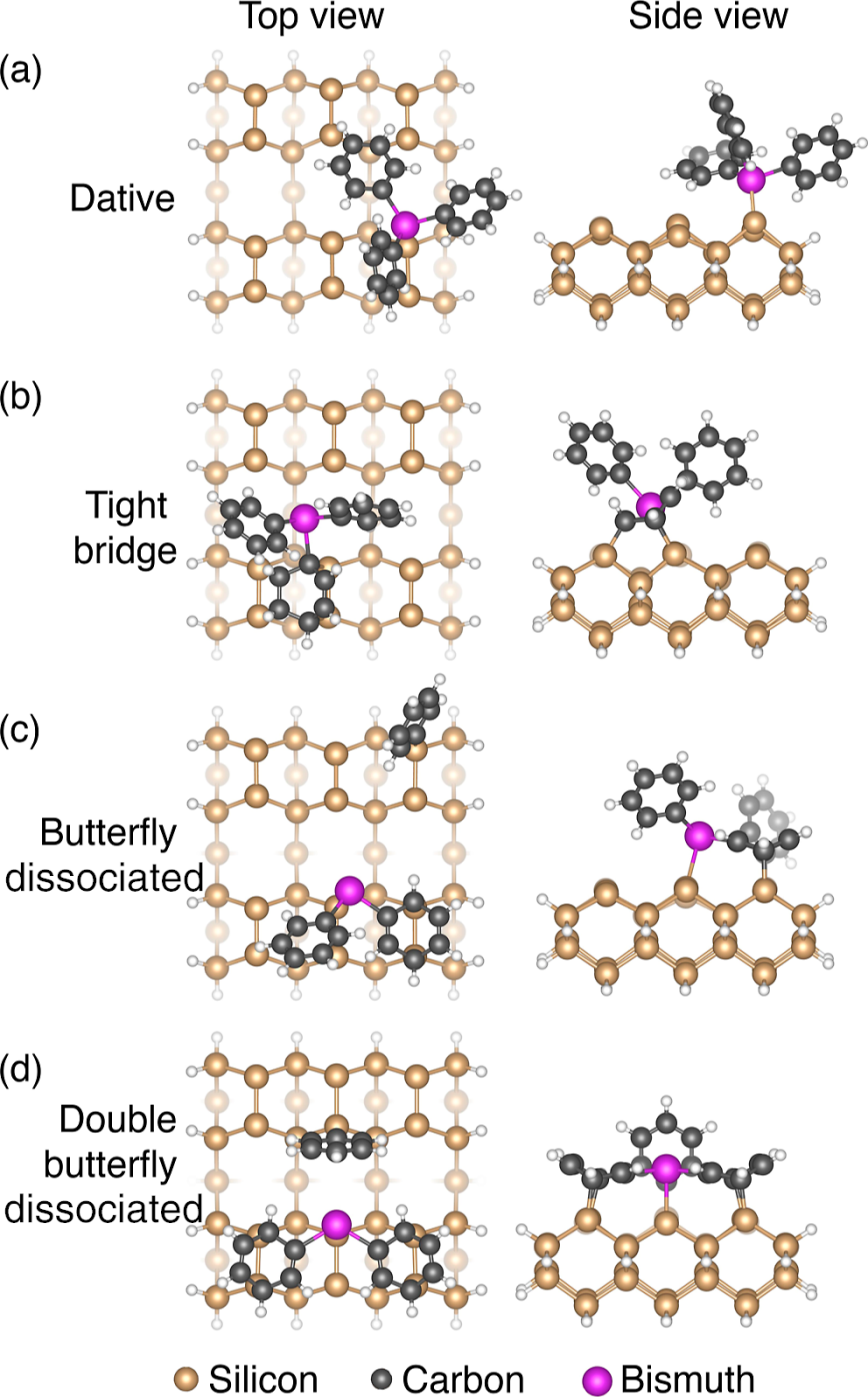
Density functional theory (DFT) geometry optimized structures for different adsorption and dissociation configurations of TPB on Si(001). (a) Datively bonded TPB attached to the surface via only the single dative Bi–Si bond; (−0.6 eV). (b) TPB molecule attached to the Si(001) surface via only one of its phenyl rings, which is bonded in the tight-bridge geometry known from the adsorption of benzene to Si(001). There is no Bi–Si bond in this structure; (−1.4 eV). (c) Dissociated TPB molecule consisting of two fragments. One fragment is bonded via both a Bi–Si bond and two C–Si bonds of one phenyl ring in a butterfly configuration. The second fragment is one phenyl ring that is bonded to the neighboring dimer row; (−4.1 eV). (d) Similar to (c), but now the first fragment is attached via both of its phenyl rings; (−4.4 eV).

To better understand the adsorption of TPB on Si(001), we have performed a DFT calculation survey of configurations involving attachment of TPB to Si(001) in various states of Si–Bi and Si–C bonding and phenyl dissociation. The large size of the TPB molecule leads to a very large number of possible adsorption configurations with respect to the surface lattice, and the flexibility of the molecule and possibilities for phenyl dissociation further increases the configuration space of possible adsorption structures. As such, a definitive assignment of a structure to every feature observed by STM is not practical. Instead, we focus here on presenting an overview of the basic types of bonding we anticipate in the adsorption structures. Figure 2 shows the geometry-optimized structures for four different main possible configurations of TPB on the Si(001) surface. In Figure 2a, we show a datively bonded TPB molecule attached to the surface through a single Bi–Si bond. With a shallow binding energy of −0.6 eV, this configuration is likely to be unstable with respect to the formation of additional Si–C bonds. However, it illustrates how the molecule may first attach to the surface in the case where it lands with the Bi atom closest to the surface.

The shape of the TPB molecule makes it more likely that the adsorbate will interact with the surface first via a phenyl group and in this case, we can anticipate the formation of Si–C bonds analogous to the bonding of benzene to Si(001).^32^Figure 2b depicts one such possibility, where a TPB molecule is bonded to the Si(001) surface via one of its phenyl rings in a tight-bridge geometry.^32^ The pair of Si–C covalent bonds produces a binding energy for this structure of −1.4 eV. However, as with the dative bonded structure in 2a, it is likely that this structure will further stabilize via the formation of additional bonds to the surface, given the large size and flexibility of TPB.

Figure 2c,d shows examples of more complex bonding involving the formation of both Si–Bi and Si–C bonds and also the dissociation of phenyl rings away from the initial adsorbate. In Figure 2c, one fragment is bonded to the surface via a Bi–Si bond and two C–Si bonds of a phenyl ring in a butterfly configuration, while the second fragment, consisting of a single phenyl ring, is bonded to a neighboring silicon dimer row (−4.1 eV). Figure 2d is similar to Figure 2c, but with the first fragment attached to the surface via both of its phenyl rings (−4.4 eV). Such configurations, or closely related variations of them, are likely candidates to explain the features observed in our STM images; in particular, the structure shown in Figure 2d bears striking similarity to the M3 STM image feature shown in Figure 1e and the structure shown in Figure 2c, or a closely related variation, might reasonably explain features such as those shown in Figure 1f,g.

The large size and many degrees of freedom of the TPB molecule prohibit an exhaustive DFT investigation of its bonding configurations and reaction pathways on Si(001) at this time. For example, we have not considered structures involving deprotonation of the phenyl rings because we are not aware of any examples where such deprotonation reactions from phenyl ring structures have been definitively shown to explain experimental observations of metalorganic or organic phenyl-containing adsorbates on Si(001).^35^ However, we note that some DFT modeling has suggested beta hydrogen dissociation may explain STM observed biphenyl adsorption configurations on Si(001)^36^ and as such the many adsorption configurations involving beta hydrogen desorption may be of interest for future work.

Nevertheless, Supporting Information, Figures S2–S27 present a wide range of representative structures that we have investigated in this work. These structures we group into four broad categories, ordered via increasing stability: dative bonding only; dative bonding plus phenyl bonding or phenyl bonding only; dative bonding plus phenyl dissociation; and dative and phenyl bonding with also phenyl dissociation. The computational results presented here are consistent with the interpretation of molecularly and dissociatively adsorbed TPB leading to the structures we observe on the room-temperature dosed Si(001) surface. In future work, following extensive analysis of all possible surface reaction pathways, simulated STM images based on DFT results could be beneficial for providing additional insights into the complex adsorption configurations.

### Thermal Transformations of TPB Followed by STM

**Figure 3:**
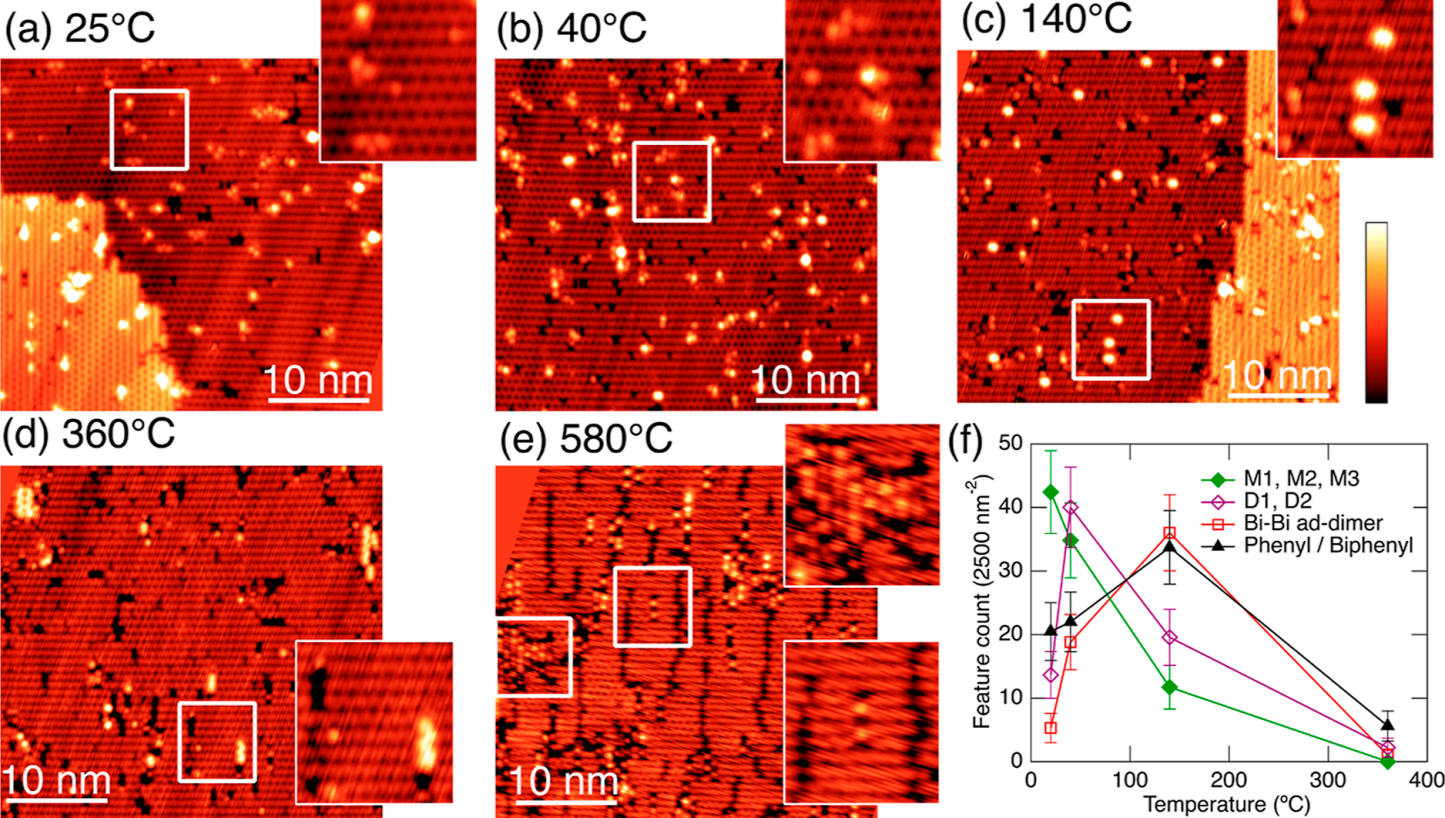
STM images of a Si(001) surface that was exposed to TPB for 1 s at room temperature and subsequently annealed to successively higher temperatures. (a) Room-temperature exposure to TPB. The inset shows a higher resolution image of an area containing an M1 and M3 feature, two adsorbed benzene, and a single Bi–Bi ad-dimer. (b–e) after annealing to 40, 140, 360, and 580 °C, respectively. Insets show enlargements of certain regions of the surfaces as indicated. (f) Plot of the feature count vs temperature. Some of the features have been grouped into categories: molecular (M1, M2, and M3) and dissociated (D1 and D2).

To further confirm our interpretation of the room-temperature data and to investigate the incorporation of bismuth into the surface, we have studied the TPB-dosed Si(001) surface as a function of annealing temperature. A clean Si(001) surface was exposed to TPB for 1 s and then annealed for 5 min at consecutively higher temperatures. After each anneal, the sample was cooled to 78 K and imaged with STM. Images of the surface after dosing at room temperature and subsequent annealing to 40, 140, 360, and 580 °C are shown in Figure 3a–e. Figure 3f shows a graph where we have counted all the features that occur on the room-temperature dosed surface and plot how the number of these features changes with each anneal.

Significant changes are observed after each successive anneal. The room-temperature surface (Figure 3a) displays the characteristics already described in Figure 1b, with the molecularly adsorbed TPB features M1, M2, and M3 being dominant. After annealing to 40 °C (Figure 3b), the dissociated features D1 and D2 become the most common, although there still exists a significant number of undissociated M1, M2, and M3 features. We also see an increase in the number of Bi–Bi ad-dimers and isolated phenyl groups. After annealing to 140 °C, the most common features on the surface are Bi–Bi ad-dimers, and the second most common features are attributed to dissociated carbon species, benzene/phenyl and biphenyl; the number of adsorbed and dissociated TPB (M1, M2, M3, D1, and D2) are noticeably reduced at this temperature. Other than these changes in the relative abundance of each type of feature, there are no other qualitative changes to the surface observed following annealing up to 140 °C. Thus, the changes we observe after annealing to 40 and 140 °C are consistent with our interpretation that features observed in the room-temperature dosed STM images are the result of thermally activated surface dissociation of TPB.

In contrast, the surface is strongly modified from its room-temperature appearance after annealing to 360 °C. The adsorbate features observed at the lower temperatures are almost completely absent. In their place, we find a surface that exhibits short zig-zagged row protrusions running perpendicular to the underlying dimer row direction and an increase in the density of surface vacancy defects that have also begun to align into short defect chains. This is a common feature of the Si(001) surface dosed with phosphine and arsine and subsequently annealed;^11,28^ the dopant atoms are incorporated into the top layer of the silicon crystal and silicon atoms are ejected onto the surface in the process. These ejected silicon atoms diffuse and nucleate to form short epitaxial chains.^11,28^ Thus, the zig-zagged rows can be readily attributed to epitaxial silicon dimer rows and their appearance on the surface after annealing to 360 °C is a strong indicator that bismuth atoms have incorporated into the Si(001) surface,^11,28^ ejecting silicon atoms onto the surface in the process.

Upon annealing to 580 °C, we observe that the short defect chains present on the surface after annealing to 360 °C have significantly enlarged and become aligned, resembling the initial formation of a 2 × *n* reconstruction known to occur due to surface strain relaxation after trace metal contamination of silicon.^37,38^ Previous studies of Si(001) surfaces exposed to bismuth through evaporation and subsequently annealed to 500 °C also reported the observation of a 2 × *n* surface reconstruction using low energy electron diffraction measurements.^39^ Moreover, similar defect chains have been reported in degenerate phosphorus-doped Si(001) samples, which could only be removed through extended annealing to deplete phosphorus atoms from the near-surface region.^40^ These findings suggest that annealing to 580 °C leads to the diffusion of bismuth beneath the surface, and the resulting strain relaxation at the surface is facilitated by the formation of defect chains in a pattern similar to the initial stages of formation of a 2 × *n* surface reconstruction.

In addition to the extended defect chains, we detect small regions of a 4 × 4 reconstruction on our 580 °C annealed surface. It is known that a 4 × 4 reconstruction can form due to both bismuth^41^ and carbon^42^ impurity contamination after annealing to approximately 600 °C. Thus, while we cannot definitively attribute the 4 × 4 reconstruction entirely to either bismuth or carbon contamination, the absence of any other features attributable to carbon on the surface suggests that the dominant factor in the 4 × 4 reconstructed regions is carbon contamination.

### Thermal Transformations of TPB Followed by XPS

**Figure 4:**
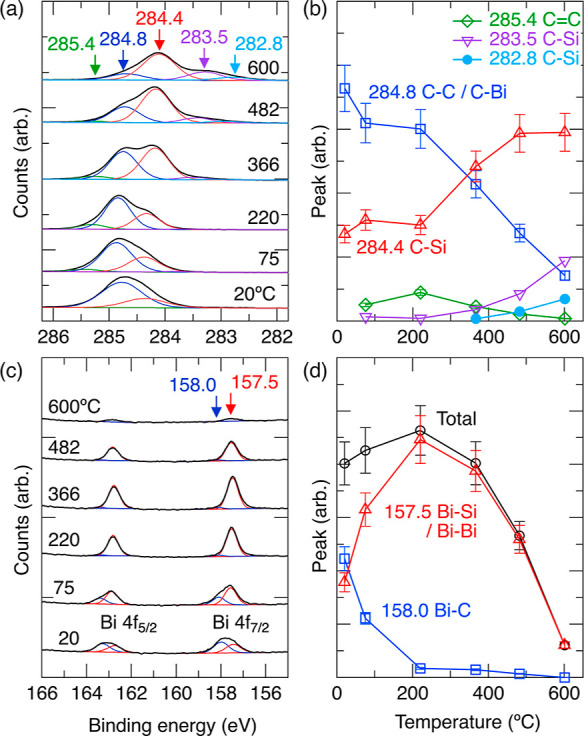
XPS measurements of Si(001) surfaces exposed to TPB for 15 s and then subsequently annealed to 75, 220, 366, 482, and 600 °C, as indicated. (a) C 1s spectra have been fit with peaks at 282.8, 283.5, 284.4, 284.8, and 285.4 eV. (c) Bi 4f spectra have been fit with peaks at 157.5 and 158.0 eV. (b,d) Plots of the variation of the peak areas with anneal temperature. The total area is also shown.

We can gain further chemical insights into the surface structures using XPS. For XPS analysis, we have exposed the sample to TPB for 15 s in order to have a coverage that is low enough for the surface to exhibit sub-monolayer coverage while also providing a good signal-to-noise ratio for XPS measurements. Although the coverage in this set of investigations is necessarily higher than in the STM work presented above, the chemical transformations at this submonolayer coverage are expected to follow the same overall trends as observed in STM measurements. Figure 4 summarizes the temperature-dependent XPS investigations of this sample. The C1s spectra are shown in Figure 4a. Each spectrum was acquired at room temperature after the sample was annealed to the temperature indicated on the plot. Figure 4b shows a plot of the relative intensities of each C 1s peak as a function of temperature between room temperature and 600 °C. We fit these spectra with two main components at 284.4 and 284.8 eV, a much smaller peak at 285.4 eV, and two additional features at 283.5 and 282.8 eV, which can only be clearly observed at higher temperatures. These peaks clearly fall into the same energy range as has been observed earlier following deposition of other carbon-containing compounds on Si(001), e.g., acetophenone^43^ and C_60_ on Si(001).^44^ These binding energy values are also very similar to observations of C–C, C=C, and C–Si peaks with the XPS reported previously.^45^ As the substrate is annealed to successively higher temperatures, we find the 284.4 eV peak, associated with Si–C bonds, grows at the expense of the 284.8 eV peak. The emergence of two additional peaks at 283.5 and 282.8 eV is recorded starting at the 366 °C anneal, and these continue to increase in intensity up to our hottest anneal (600 °C).

The Bi 4f spectra after each successive anneal are also recorded and are shown in Figure 4c. The relative intensities of the observed peaks are plotted in Figure 4d. In this case, only two peaks corresponding to the Bi 4f7/2 are observed at 157.5 and 158.0 eV (as well as their spin–orbit split counterparts). We find the two peaks have approximately equal intensities at room temperature. The 158.0 eV peak deceases in intensity nearly to zero when the sample is annealed up to 220 °C, while over the same temperature range, the 157.5 eV peak increases. Above 220 °C, the 157.5 eV peak also decreases in intensity and nearly completely disappears at our highest annealing temperature of 600 °C.

We further confirm the assignment of these XPS spectra with the aid of the DFT computational results shown in Figure 2, which indicates the main types of substrate–surface interactions that are anticipated. For these configurations, the expected positions of the C 1s features were predicted using Koopmans’ theorem. These values were calibrated by comparing the predicted and experimentally recorded positions of C 1s features for benzene using the same computational methods as for the structures investigated. The average position for the C 1s in benzene was set to 284.6 eV. This calibration resulted in an adjustment of 7.5 eV for the structures presented in Figure 2. This approach has been well documented to provide a reliable comparison between computed and experimental energies of core level energy levels in light elements and has performed especially well for N 1s and C 1s comparison.^45−48^ Intriguingly, the majority of the C 1s features expected from the initially formed structures, which are predominantly associated with carbon–carbon and carbon–bismuth interactions, are computationally forecasted to appear around 284.6 eV. Meanwhile, features that represent a variety of C–Si bond configurations are anticipated to be centered at approximately 0.2–0.3 eV lower binding energy. This appears to indicate that the features grouped around the 284.8 eV peak and around the 284.4 eV peak correspond to carbon–carbon (or unperturbed carbon–bismuth) bonds and C–Si bonds, respectively. However, not all C–Si bonds fit neatly into these classes. In particular, in a dissociated phenyl group adsorbed on a surface via a C–Si bond, the observed features are predicted to be notably different for the top three carbon atoms compared to the rest of the molecule. Correspondingly, we assign the small feature observed around 285.4 eV to the signature of an intact phenyl ring bound directly to the surface following C–Bi bond dissociation.

If the feature at 284.4 eV represents a combination of various surface-bound (C–Si) carbon species, such as for example the carbon atoms in butterfly and pedestal surface bound configurations, it would be expected to increase in intensity as the surface annealing temperature is increased. This is indeed the case. As the surface temperature increases, this feature becomes dominant all the way up to the annealing temperature of 482 °C. This observation is consistent with the assumption that more and more surface bound (C–Si) structures are formed and that these structures are more thermodynamically stable than the products of initial attachment. Concurrently with this increase, the features corresponding to structures with relatively unperturbed C–C and C–Bi configurations (284.8 eV) decrease in intensity.

At high annealing temperatures (≥366 °C), features at 283.5 and 282.8 eV appear, suggesting the formation of very tightly surface-bound carbon structures and silicon carbide.^49^ Importantly, within our uncertainty estimates, the total intensity of the carbon signal does not change with temperature, meaning that within this temperature range, all carbon atoms remain on the surface of the crystal up to 600 °C.

The temperature-dependent analysis of Bi 4f spectra provides further insights into the TPB chemistry. Bismuth 4f features at 158.0 eV quickly disappear upon annealing. Combined with the C 1s changes noted above, we propose that this feature represents Bi atoms that are not directly connected to the surface. In other words, carbon scaffolds separate the Bi atoms in these structures from the silicon surface. As the surface is annealed, these structures transform into the more stable ones, with a Bi atom in direct contact with the surface. The spectroscopic signature of these structures is a dominant feature at 157.5 eV, which remains positioned at this binding energy at higher annealing temperatures as well. The combined intensity of these two peaks remains the same within our error of measurement, up to the annealing temperatures above 482 °C, where the overall bismuth signal decreases, which appears to correspond to the diffusion of Bi into the subsurface, exactly as suggested by our STM data. It is unlikely that bismuth sublimes at such low temperatures; e.g., it is known that phosphorus desorbs from Si(001) as P_2_ at a temperature of around 800 °C, or slightly lower at very high coverages.^50−52^

## Conclusions

We have investigated the adsorption and thermal decomposition of triphenyl bismuth (TPB) on the silicon (001) surface, combining atomic resolution STM, synchrotron-based XPS, and DFT calculations. Our findings reveal that TPB adsorption at room temperature results in the formation of both bismuth–silicon and carbon–silicon bonds and that the adsorption is partially dissociative. Annealing at increased temperatures leads to complete phenyl detachment, subsurface migration of bismuth atoms, and eventually the formation of a 4 × 4 surface reconstruction ascribed to be due to the surface carbon. This work highlights the potential of using TPB dosing and surface annealing for the ultra-shallow doping of silicon with bismuth, a technique with possible applications in microphotonics and spintronics. While carbon impurities are introduced to the surface, it is worth noting that these impurities can be removed via chemical treatment or sputtering. The insights gained from this investigation contribute significantly to the understanding of chemisorption and dissociation processes on semiconductor surfaces.

## Supporting Information Available

Three-dimensional model of the cluster used in DFT calculations, binding energy plot for all structures calculated, ball and stick figures for all structures calculated, and XYZ structure files for the optimized geometries of all structures (PDF)
