# Supplementary material for: Adsorption and Thermal Decomposition of Triphenyl Bismuth on Silicon (001)
Source: J Phys Chem C Nanomater Interfaces. 2023 Aug 14;127(33):16433–41. doi: 10.1021/acs.jpcc.3c03916 (PMC10461293; doi:10.1021/acs.jpcc.3c03916)
Supplement: Supplementary file 1 — jp3c03916_si_001.pdf [file jp3c03916_si_001.pdf]

SUPPORTING INFORMATION: Adsorption and Thermal Decomposition of Triphenyl Bismuth on Silicon (001)

Eric A. S. Lundgren,[†,‡] Carly Byron,[¶,#] Procopios Constantinou,[†,‡,§] Taylor J. Z. Stock,[†,∥] Neil J. Curson,[†,∥] Lars Thomsen,[⊥] Oliver Warschkow,[†] Andrew Teplyakov,[*,¶] and Steven R. Schofield[*,†,‡]

†*London Centre for Nanotechnology, University College London, WC1H 0AH, London, UK*

‡*Department of Physics and Astronomy, University College London, WC1E 6BT, London, UK*

¶*Department of Chemistry and Biochemistry, University of Delaware, Newark, Delaware 19716, United States*

§*Paul Scherrer Institute, 5232 Villigen, Switzerland*

∥*Department of Electronic and Electrical Engineering, University College London, WC1E 7JE, London, UK*

⊥*Australian Synchrotron, ANSTO, Clayton, VIC 3168, Australia*

#*Present address: Chemical Sciences and Engineering Division, Argonne National Laboratory, Lemont, Illinois 60439, United States*

E-mail: andrewt@udel.edu; s.schofield@ucl.ac.uk

Abstract

In this Supplementary Information, we provide additional data and computational details supporting our main manuscript titled “Adsorption and thermal decomposition

of triphenyl bismuth on silicon (001)". We show STM images of triphenyl bismuth adsorbed to Si(001) and different coverages. We show a three-dimensional ball and stick model of the cluster type used for all density functional theory (DFT) calculations. We present a binding energy plot showing the energies all the structures we have computed. This plot elucidates the energetic relationships between these structures. Lastly, we provide a series of figures featuring top and side-view ball and stick structure models for all computed structures.

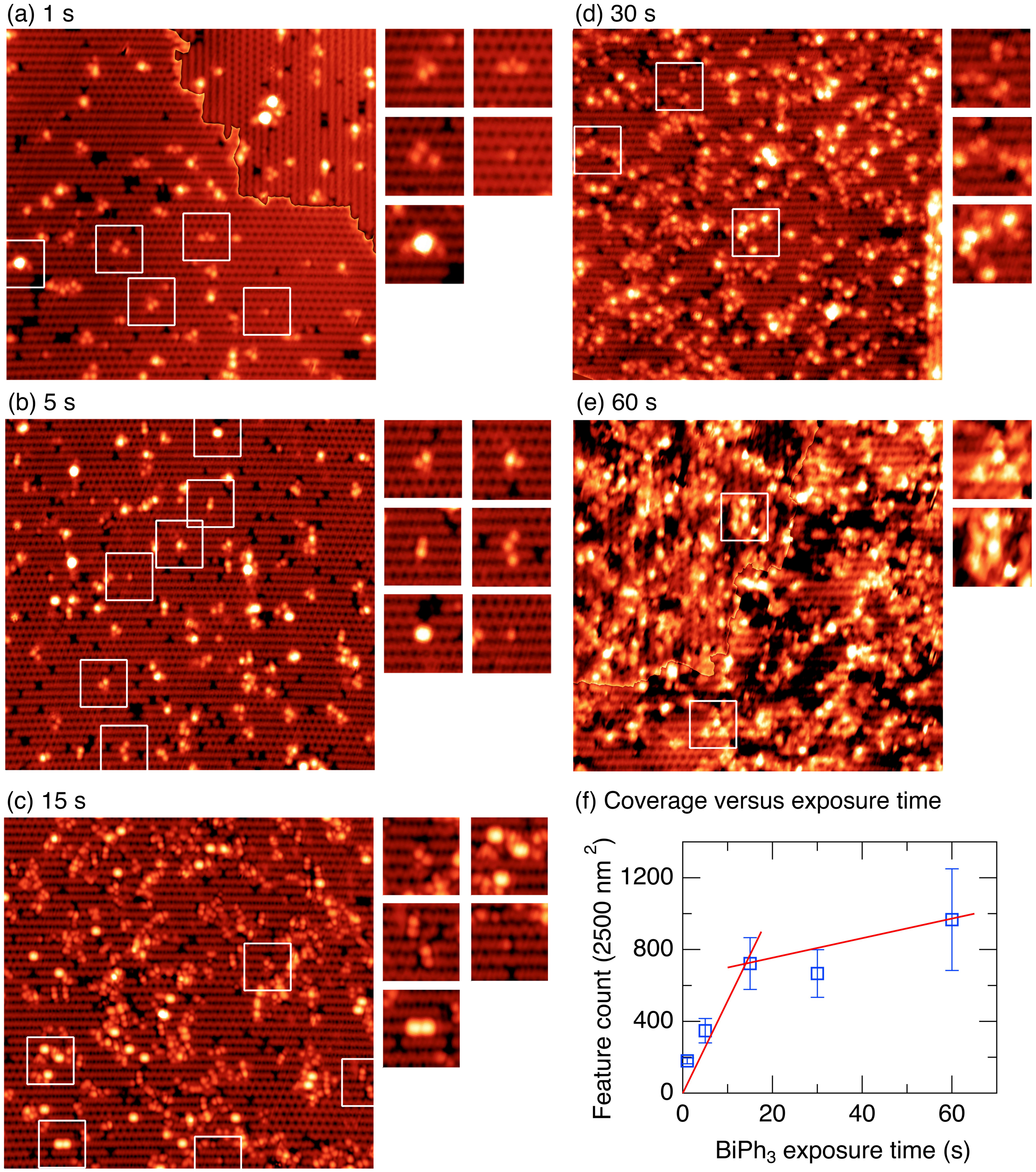

Supporting Figure S1: STM images of a Si(001) surface exposed to submonolayer coverages of TPB. The surfaces were exposed to a TPB source heated to 80°C with the silicon substrate held at room temperature for (a) 1 s, (b) 5 s, (c) 15 s, (d) 30 s, and (e) 60 s. (f) Shows a count of the number of adsorbate features identified per 2500 nm^2, indicating that the initial adsorption is linear, but that for exposure times greater than 15 s, the rate of increase in coverage slows, which can be attributed to the influence of steric interactions on adsorption. Enlargements of a selection of individual features are provided alonside each image. A step edge runs diagonally through the image (e) and the background plane subtraction has been applied separately on each terrace. Image parameters: -2 V, 30×30 nm^2 (main image), 50 pA, 78 K.

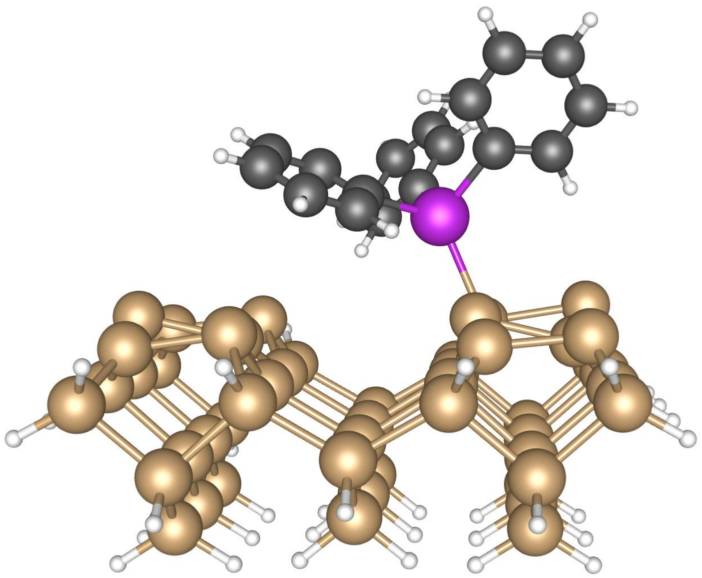

Supporting Figure S2: $Si_{49}H_{40}$ cluster model with adsorbed triphenyl bismuth molecule in a dative bonded configuration.

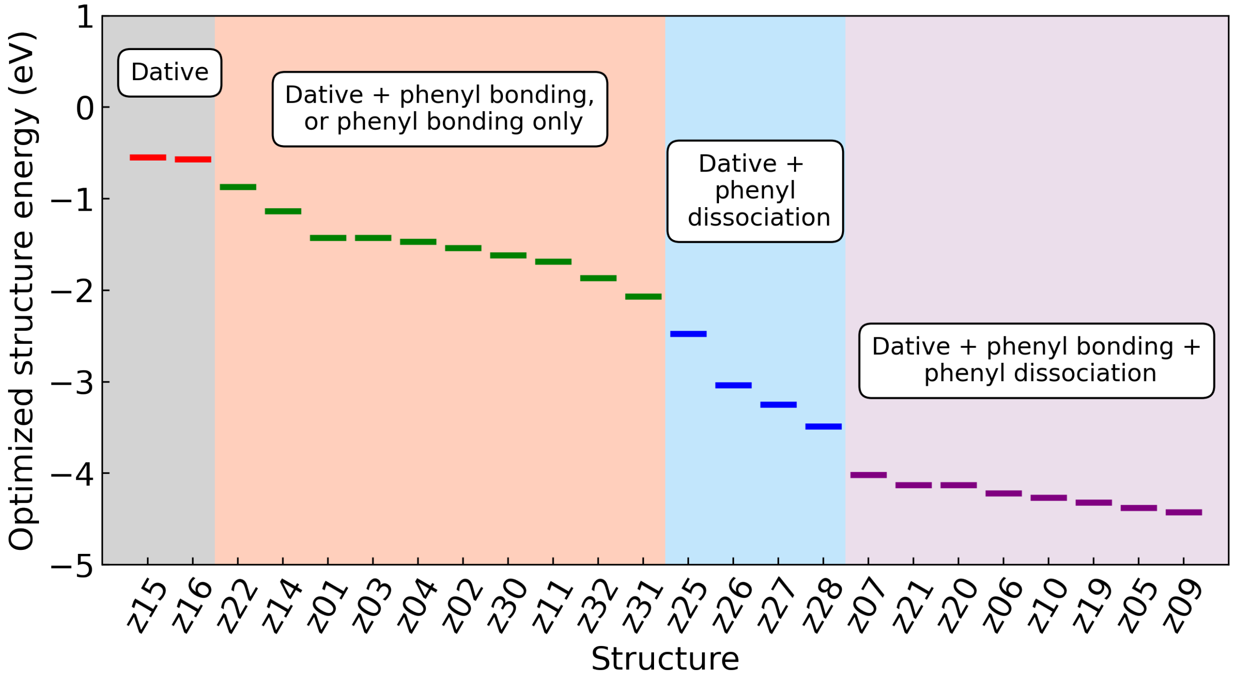

Supporting Figure S3: Comparative Energy Analysis for Different Adsorption Structures. Displayed are the binding energies corresponding to each considered structural configuration of triphenyl bismuth adsorbed on the silicon (001) surface. Each marker represents a distinct structure with its respective energy denoted in electron volts (eV). The structures are classified into four bonding categories: 'Dative', 'Dative + Phenyl Bonding', 'Dative + Phenyl Dissociation', and 'Dative + Phenyl Bonding + Phenyl Dissociation'. Each class is visually demarcated by a unique background color. The information encapsulated in this plot allows for a comprehensive understanding of the relative energetic stabilities across all explored structures.

Optimised structures

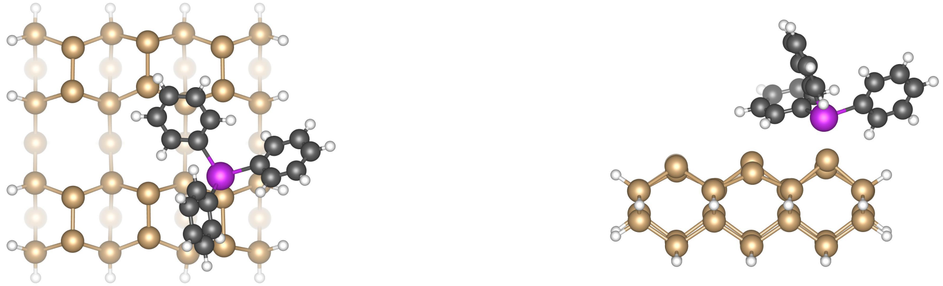

Supporting Figure S4: z15 (singlet) -0.55 eV

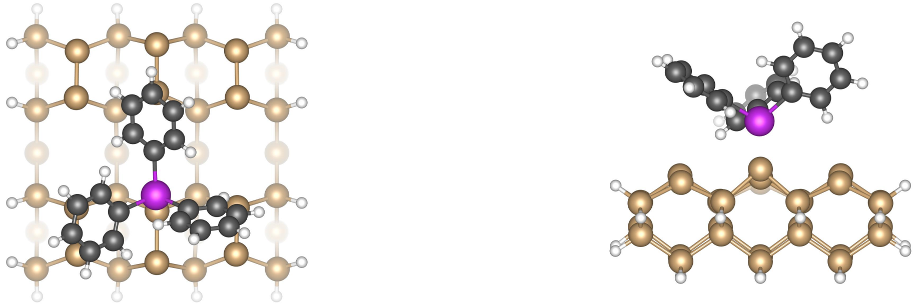

Supporting Figure S5: z16 (singlet) -0.57 eV

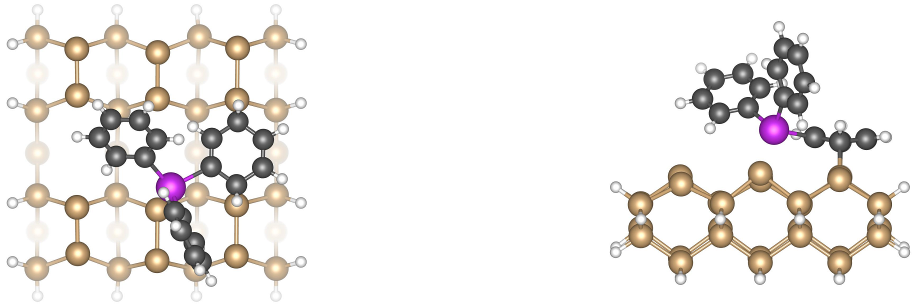

Supporting Figure S6: z22 (singlet) -0.87 eV

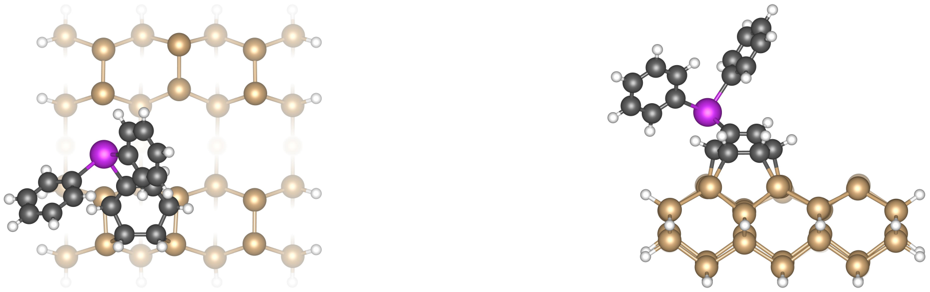

Supporting Figure S7: z14 (singlet) -1.14 eV

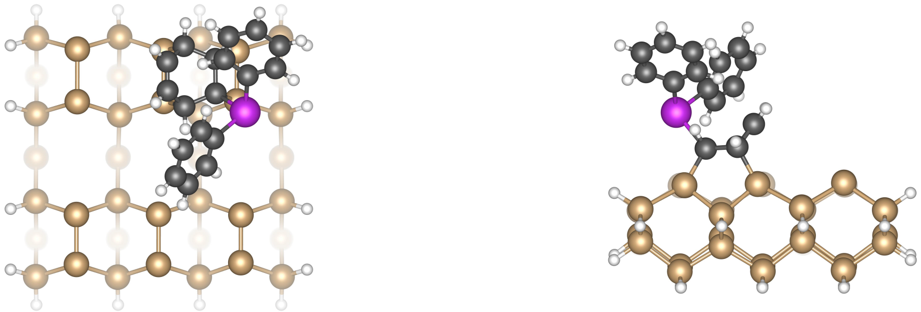

Supporting Figure S8: z01 (triplet) -1.43 eV

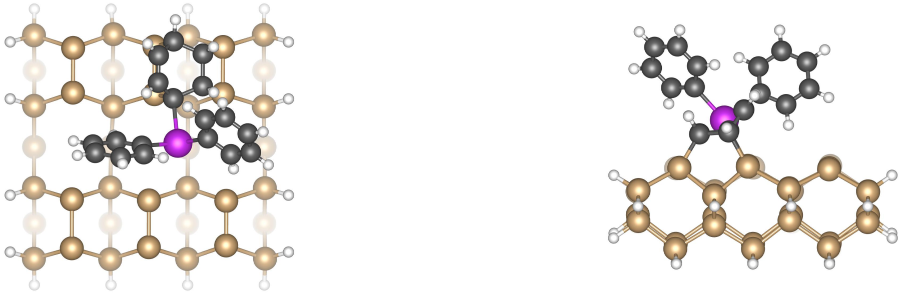

Supporting Figure S9: z03 (triplet) -1.43 eV

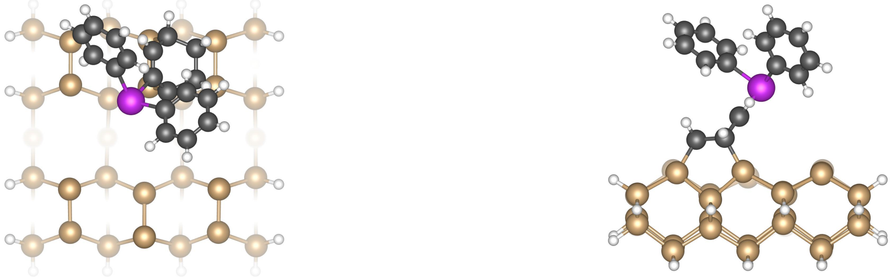

Supporting Figure S10: z04 (singlet) -1.47 eV

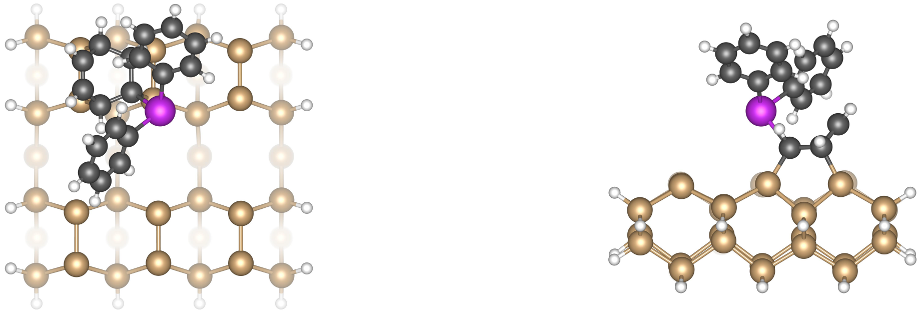

Supporting Figure S11: z02 (triplet) -1.54 eV

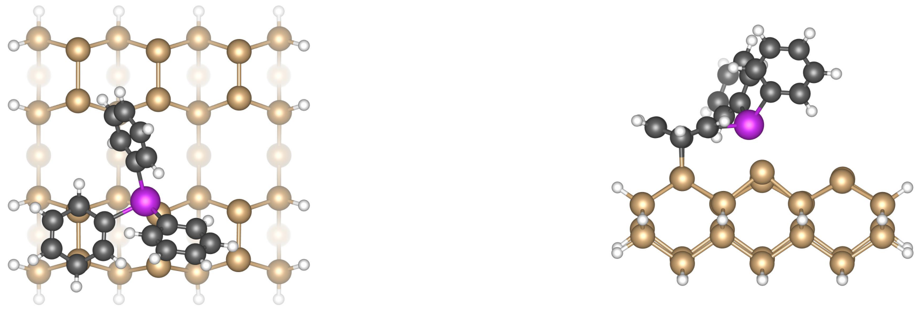

Supporting Figure S12: z30 (triplet) -1.62 eV

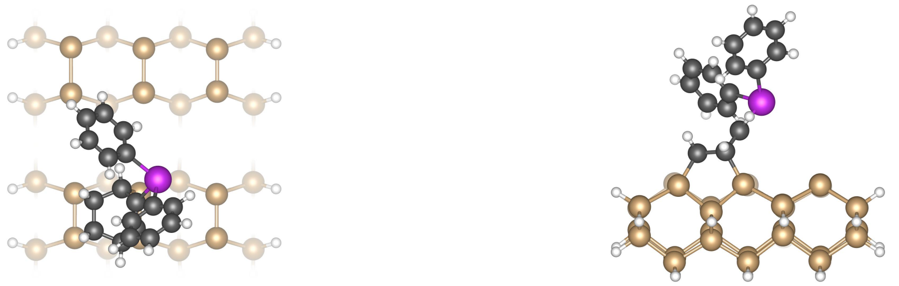

Supporting Figure S13: z11 (triplet) -1.69 eV

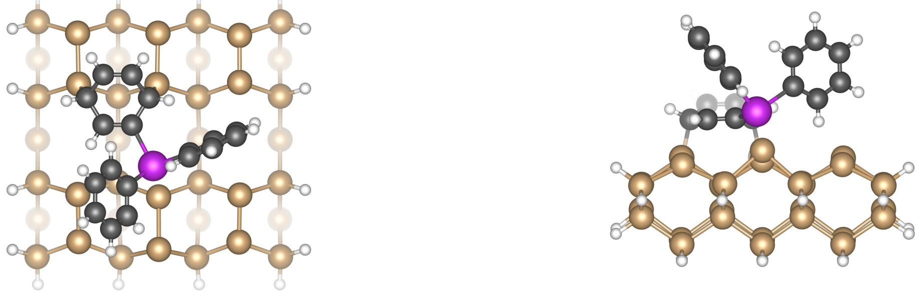

Supporting Figure S14: z32 (triplet) -1.87 eV

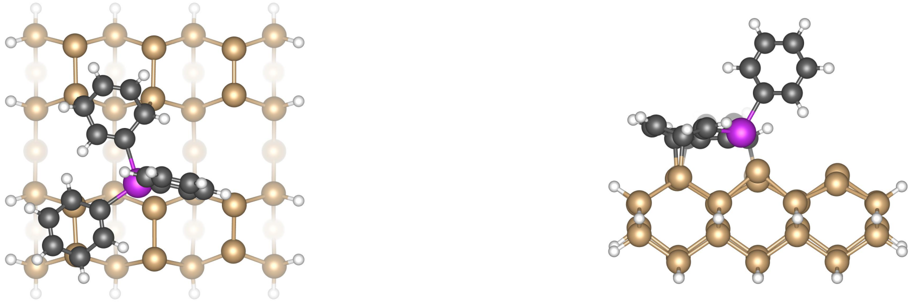

Supporting Figure S15: z31 (triplet) -2.07 eV

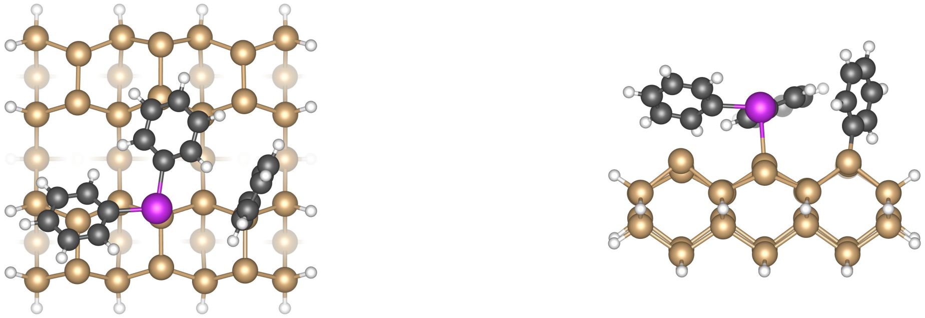

Supporting Figure S16: z25 (singlet) -2.48 eV

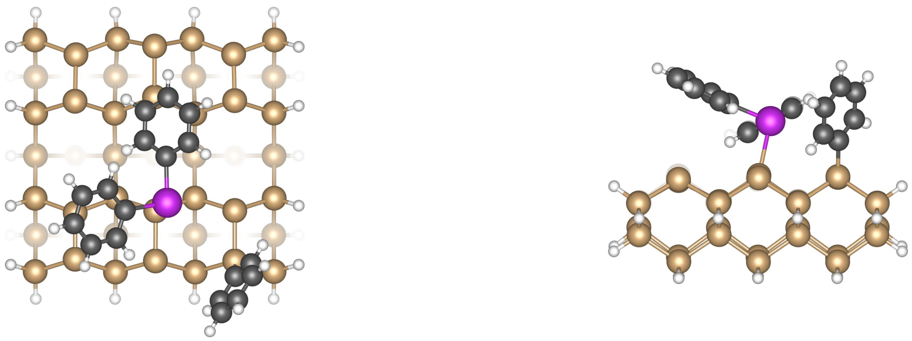

Supporting Figure S17: z26 (triplet) -3.04 eV

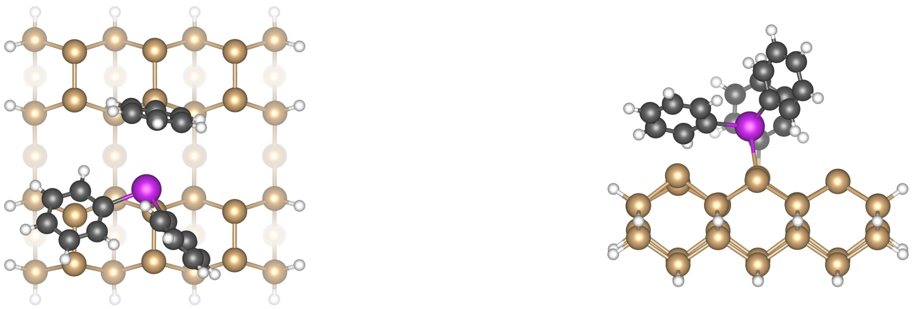

Supporting Figure S18: z27 (triplet) -3.25 eV

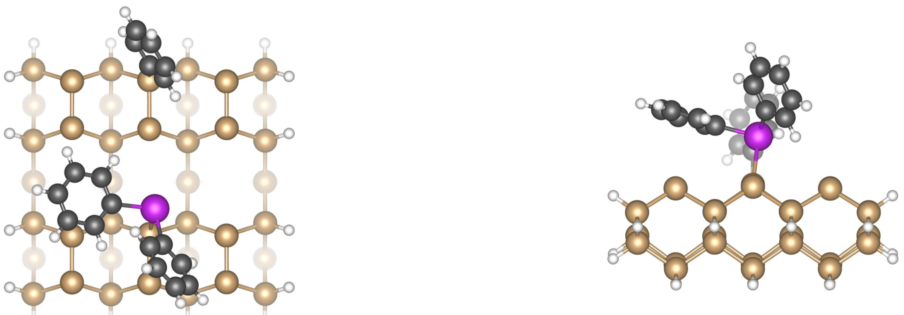

Supporting Figure S19: z28 (triplet) -3.49 eV

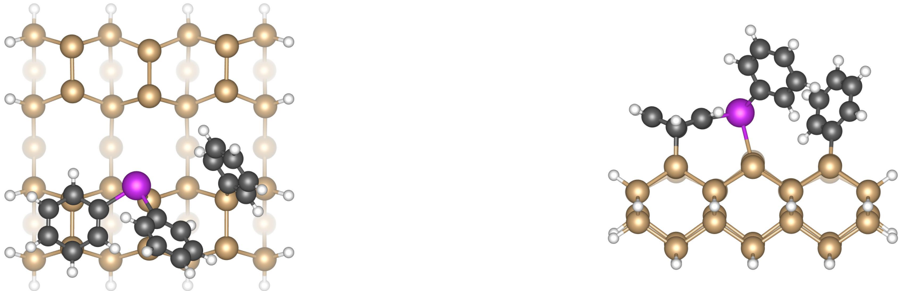

Supporting Figure S20: z07 (triplet) -4.02 eV

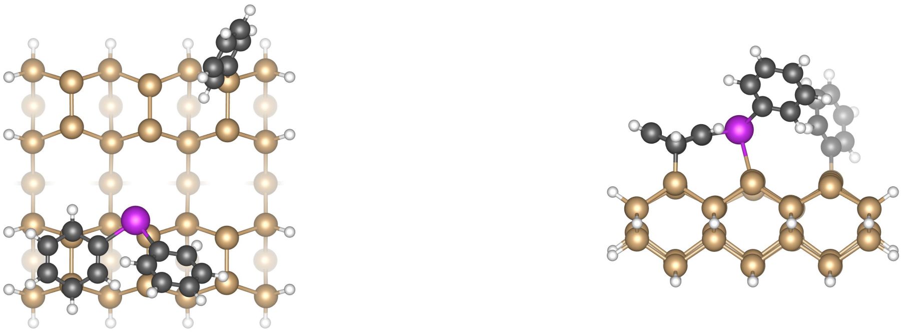

Supporting Figure S21: z21 (triplet) -4.13 eV

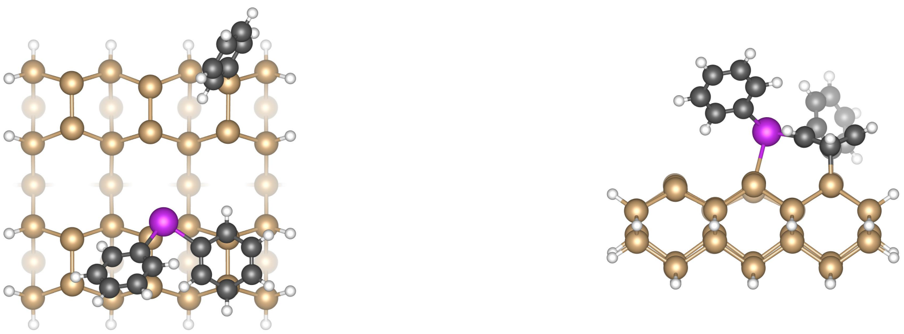

Supporting Figure S22: z20 (triplet) -4.13 eV

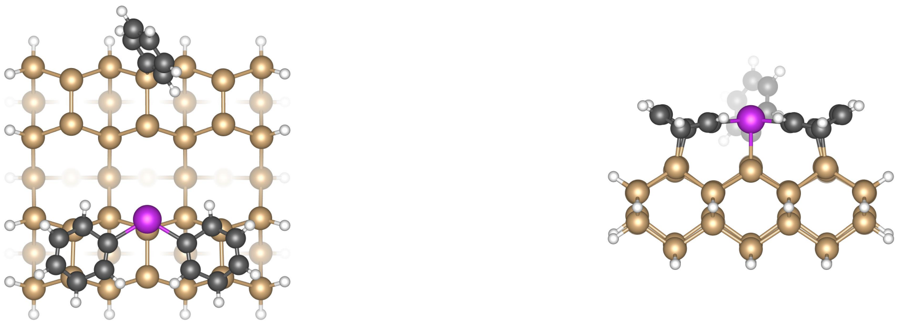

Supporting Figure S23: z06 (triplet) -4.22 eV

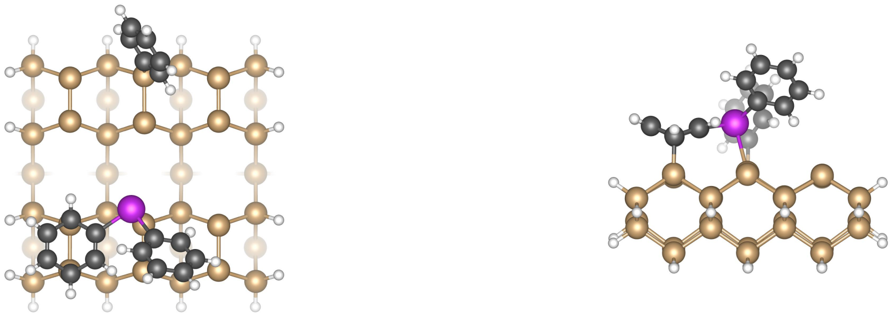

Supporting Figure S24: z10 (triplet) -4.27 eV

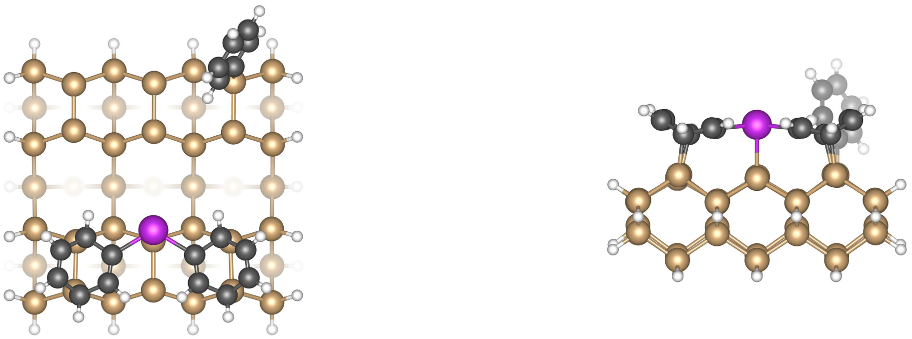

Supporting Figure S25: z19 (triplet) -4.32 eV

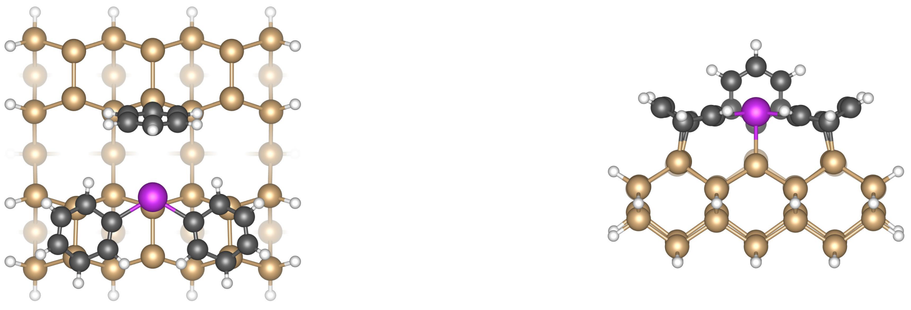

Supporting Figure S26: z05 (triplet) -4.38 eV

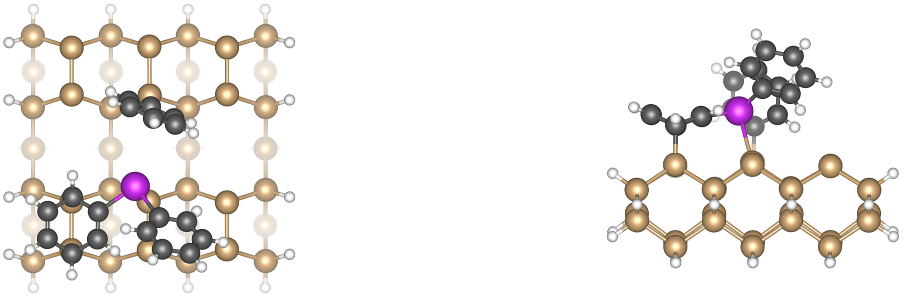

Supporting Figure S27: z09 (triplet) -4.43 eV
